# The Hole Truth: Why Do Bumble Bees Rob Flowers More Than Once?

**DOI:** 10.3390/plants13172507

**Published:** 2024-09-06

**Authors:** Judith L. Bronstein, Goggy Davidowitz, Elinor M. Lichtenberg, Rebecca E. Irwin

**Affiliations:** 1 Department of Ecology and Evolutionary Biology, University of Arizona, Tucson, AZ 85721, USA; 2 Department of Entomology, University of Arizona, Tucson, AZ 85721, USA; goggy@arizona.edu; 3 Department of Biological Sciences and Advanced Environmental Research Institute, University of North Texas, Denton, TX 76203, USA; elinor.lichtenberg@unt.edu; 4 Department of Applied Ecology, North Carolina State University, Raleigh, NC 27695, USA; reirwin@ncsu.edu; 5 Rocky Mountain Biological Lab., Crested Butte, CO 81224, USA

**Keywords:** pollination, nectar robbing, *Corydalis caseana*, cheating, mutualism, bumble bees, foraging, nectar

## Abstract

Primary nectar-robbers feed through holes they make in flowers, often bypassing the plant’s reproductive organs in the process. In many robbed plants, multiple holes are made in a single flower. Why a flower should be robbed repeatedly is difficult to understand: a hole signals that a nectar forager has already fed, which would seem likely to predict low rewards. We tested three explanations for this pattern in *Corydalis caseana* (Fumariaceae), a bumble bee pollinated and robbed plant: (1) multiple holes appear only after all flowers have been robbed once; (2) individual foragers make multiple holes during single visits; and (3) it is more profitable for bees to rob older flowers, even if they have already been robbed. We tested these hypotheses from 2014 to 2016 in a Colorado, USA population using data on robbing rates over time, floral longevity, nectar accumulation in visited and unvisited flowers, and the accumulation of robbing holes across the life of flowers. Multiple holes were already appearing when two-thirds of flowers still lacked a single hole, allowing us to reject the first hypothesis. The second hypothesis cannot offer a full explanation for multiple robbing holes because 35% of additional holes appeared in flowers one or more days after the first hole was made. Repeated sampling of bagged and exposed inflorescences revealed that flowers filled at a constant rate and refilled completely after being drained. Consequently, young flowers are of consistently low value to foragers compared to older flowers even if they had previously been robbed, consistent with the third hypothesis. While further studies are needed, these results offer a simple explanation for the paradoxical clustering of nectar-robbing damage in this and possibly other plant species.

## 1. Introduction

The study of mutually beneficial interactions between different species, or mutualism, has become a significant growth area in biology [1]. Interest has coalesced around a small set of conceptual questions. Under what conditions can organisms profit from associating with a different species, and when do the costs of these associations outweigh their benefits? Which mutualisms are specialized, generalized, obligate, and facultative, and why do these patterns exist? When and how do mutualisms structure ecological communities? Finally, how do mutualisms emerge over evolutionary time, and what forces lead to their decay? With regard to the latter question, a dominant theme has been “cheating”—diverse activities of individuals or species that benefit from the rewards and services mutualists exchange without offering any in return. By one analysis, the study of cheating is addressed in almost one-third of recent papers on mutualism [2]. A key question, whether cheating must be deterred or punished if mutualism is to persist, is hotly debated [2,3,4,5,6]. In this context, it is intriguing that individuals that fail to reciprocate within mutualisms are ubiquitous in nature, with relatively few convincing examples yet identified of effective “sanctions” against these behaviors [2,5,7]. The mismatch of theory and data suggests the need to further explore cheating in nature. To date, however, few mutualist–cheater interactions have been studied in depth, with the best data coming from laboratory systems rather than field settings e.g., [8,9,10].

The most thoroughly documented form of mutualistic cheating is floral larceny [11], a set of tactics in which foragers obtain nectar often without picking up or depositing pollen in the process. The best-documented larcenists are small insects, prominently but not exclusively bees, and birds [11]. Three forms of larceny have been identified [12]. Nectar thievery takes place when a forager enters the flower and consumes nectar without contacting the stigmas and anthers. Primary nectar robbing involves making a hole or slit near the base of a flower and consuming nectar through it; secondary nectar robbing involves re-use of such a hole. These different foraging tactics, all of them presumably beneficial to foragers themselves but some of them detrimental to the plant, can be combined in diverse ways. A flower-feeding species might always act as a larcenist of one plant species, but as a “legitimate visitor” (one that visits a flower in a way that effects pollen transfer, i.e., as a mutualist) in another. Alternatively, some individuals within a species might always be larcenists whereas others always act mutualistically. Finally, individuals may use more than one foraging tactic, combining the different forms of larceny with mutualistic visitation in plant-species-specific ways. All of these combinations have been well documented in recent years [13,14,15,16,17].

Although floral larceny is highly complex and system specific, several strong patterns have emerged. First, there is little evidence that plants have evolved effective means to deter it. In part, this is likely related to the fact that the cost to plants of being cheated is often negligible, e.g., [18,19]. Indeed, in some cases, being robbed appears to be beneficial [20,21]. Second, new perspectives are being gained by taking the forager rather than plant perspective. That is, instead of treating the phenomena as “cheating”, “larceny”, “robbing”, “thievery”, and so on—addressing the phenomena as if it is breaking a contract—one can ask what the costs and benefits of these behaviors are for the foragers themselves, e.g., [14,15,16,17,22,23,24]. Recent studies taking this perspective have revealed, for instance, that in some cases pollination rather than nectar-robbing is the most nutritionally profitable tactic, and that foragers may retain a given tactic for a prolonged period, prioritizing the reduction of search and handling times over the exploration of alternatives [14]. These phenomena are unexpected from current theory, which posits that there is a so-called temptation to cheat (i.e., cheating is always prioritized over cooperation), that cheating always pays, and that the cheated partner always suffers.

Here, we explore another empirical observation that does not align with current theory. In some nectar-robbed species, individual flowers commonly exhibit more than one robbing hole. Why a single flower should be primaryrobbed repeatedly is difficult to understand: a hole signals that a nectar forager has already fed, perhaps very recently. This would seem likely to indicate to the forager that nectar is low in abundance or absent entirely. 

We used field observations and simple experiments to explore this phenomenon. We focused on a well-studied bumble bee pollination and robbing system in Colorado, USA, centered on the plant *Corydalis caseana* (Fumariaceae). We tested three hypotheses. First, multiple holes might start accumulating in flowers only after primary robbers have attacked every flower in the population. Since previous studies have found that primary-robbing bumble bees are reluctant to switch foraging tactics (e.g., to reuse an existing hole rather than making a new one), the prediction is that, if these foragers choose to continue foraging in the same population (i.e., neither switching to a different plant species nor departing to find *C. caseana* elsewhere) they will ultimately be forced to add new holes to already-robbed flowers to extract nectar. Second, individual foragers might make multiple holes during a single visit to a flower. Third, the dynamics of nectar replenishment and accumulation of robbing holes may make older, robbed flowers more profitable for primary robbers to visit than younger ones that have never been attacked.

We argue that understanding the surprising phenomenon of multiply robbed flowers requires taking the less-adopted perspective on cheating: the options faced, and choices made, by the cheater itself. This approach can thus reveal new insights, challenging the stereotypical way that cheating has been treated in the ecological literature.

## 2. Results

### 2.1. General Patterns of Floral Longevity, Nectar Secretion, and Robbing Rates

**Figure 1:**
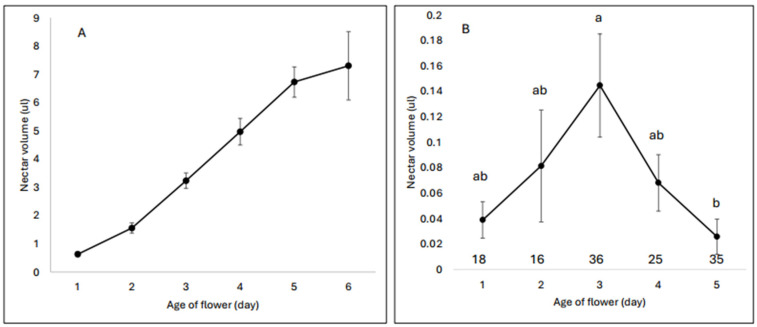
Nectar volumes in flowers of known age. Sample sizes range from 7 flowers/day–44 flowers/day. We used day means (±SEM) to calculate the best fit models, even though the data are shown without these models. (**A**) Volumes in bagged flowers allowed to accumulate nectar for the shown number of days. The line is best described by a sigmoid curve (BIC = −4.75, R2 = 0.998). (**B**) Volumes in open flowers of known age (note the difference in scale). The best fit model is a quadratic polynomial (BIC = −19.6, R2 = 0.81). Significance among days in 1B was tested with a non-parametric Wilcoxon/Kruskal–Wallis rank sum test (*p* = 0.0071), and a post hoc test among days using a Tukey–Kramer HSD (alpha = 0.05) as indicated by the lower-case letters above the symbols. Sample sizes for each day are above the abscissa.

*Corydalis caseana* flowers typically persist on the plant for 4–5 days. Sampling flowers of known ages that had been bagged and sampled once at a known age revealed that nectar is provisioned each day: the rate of nectar provision accelerates after a flower is one day old, then decelerates after 3.5 days (Figure 1A; see also Figure 5 in [25]). Thus, if flowers are entirely unvisited, the youngest flowers would be the least profitable for a nectar forager. The pattern in flowers that had been exposed to visitors was quite different (Figure 1B): the oldest and youngest flowers held the least nectar, with highest volumes present in middle-aged flowers. 

**Figure 2:**
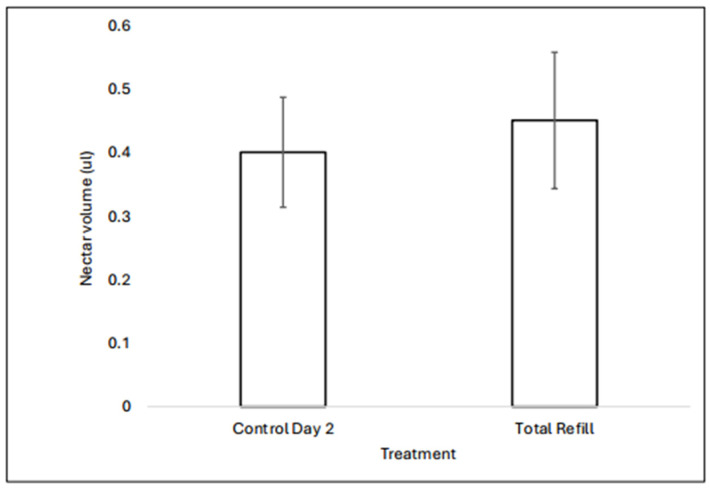
Nectar refilling dynamics. Control Day 2 flowers were bagged before opening, allowed to open in the bag, then sampled for nectar with a 2 μL capillary tube after two days. Total Refill flowers were similarly bagged before opening, but nectar was drained twice, on Day 1 and Day 2; the total amount of nectar they produced is shown. The volumes were not statistically different (two-sided *t*-test assuming unequal variances, t = 0.327, df = 36.36, *p* = 0.7458), indicating that flowers completely fill with nectar once drained.

Once drained, flowers refill with nectar. A comparison of control flowers (n = 20) containing two days’ worth of accumulated nectar with flowers (n = 20) drained and measured on both the first and second day showed no significant difference in nectar volume (two-sided *t*-test assuming unequal variances, t = 0.327, df = 36.36, *p* = 0.7458; Figure 2).

**Figure 3:**
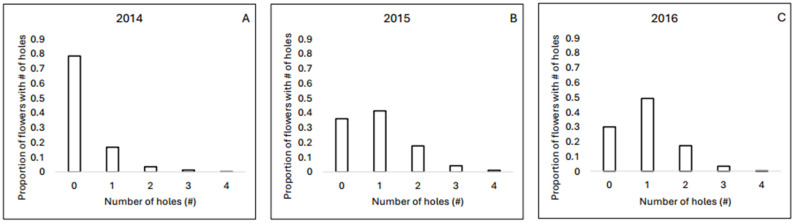
The proportion of holes in haphazardly selected *C. caseana* flowers in (**A**) July of 2014 (n = 4939 flowers), (**B**) 2015 (n = 931 flowers), and (**C**) 2016 (n = 1804 flowers).

Nectar robbing was frequent in the population (Figure 3). The percentage of flowers with at least one robbing hole was 22% in 2014 (n = 4939 flowers), 64% in 2015 (n = 931 flowers), and 70% in 2016 (n = 1804 flowers). Among the robbed flowers, 22% in 2014, 36% in 2015, and 30% in 2016 exhibited more than one and up to four holes. 

Comparing nectar volumes in robbed vs. unrobbed flowers on single dates of sampling, we found variable results by date. Unrobbed flowers sampled on 26 July 2016 contained on average about eight times more nectar than robbed flowers (one-sided *t*-test assuming unequal variances, t = 2.87, n = 117, df = 27.7, *p* = 0.0039; mean ± SEM, robbed: 0.041 ± 0.012 μL, n = 89, unrobbed: 0.343 ± 0.104, n = 28). In contrast, there was no significant difference between nectar volumes in robbed and unrobbed flowers on 10 July 2015 (one-sided *t*-test assuming unequal variances, t = 0.74, n = 96, df = 39, *p* = 0.2308; mean ± SEM, robbed: 1.29 ± 0.352 μL, n = 26, unrobbed: 1.57 ± 0.18, n = 70). Although a very small data set, these results do suggest that robbed flowers are not always less profitable than unrobbed ones.

### 2.2. Tests of Hypotheses

**Figure 4:**
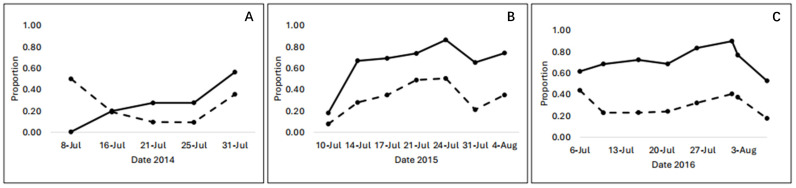
Proportion of sampled flowers on each date that were robbed (solid line) and the proportion of these that had more than one robbing hole (dashed line) in (**A**) 2014, (**B**) 2015, and (**C**) 2016.

Hypothesis 1 (multiple holes start accumulating in flowers only once primary robbers have attacked every flower in the population once) can be rejected. In each year, multiple holes were already appearing frequently even when two-thirds of flowers had not been robbed a single time (Figure 4). Indeed, in the year when less than a quarter of all flowers were robbed at all (2014), 22% of the robbed flowers had more than one hole. Unrobbed flowers were present on every flowering stalk (Bronstein et al., unpubl. data), suggesting that floral visitors encounter them regularly. These data indicate that bees are primary robbing previously robbed flowers when unrobbed ones are readily available. 

We can also reject Hypothesis 2, which posits that multiple holes are attributable to the actions of individual bees during single flower visits. If this had been the case, then all flowers with multiple holes would have acquired them on single census dates using our conservative data. In fact, 36.4% of all second, third, and fourth holes appeared in flowers one or more days after the first hole appeared (Table 1). While not ruling out the possibility that *some* visitors make multiple holes in the course of a single foraging visit, it does allow us to reject the hypothesis that this is a complete explanation for the presence of multiple holes.

Hypothesis 3 conjectures that the dynamics of nectar replenishment and accumulation of robbing holes make older flowers, even if robbed, more profitable for primary robbers to visit than younger ones that have never been attacked. In combination, multiple pieces of evidence suggest that this may indeed be the case. First, older flowers can be exceedingly valuable to visit. If they have not been visited previously, these flowers will contain very high nectar volumes (Figure 1A); if a visit (robbing or legitimate) took place early in their lives, nectar replenishment dynamics (Figure 2) suggest that they will still contain more nectar than young flowers. Second, flowers of known age exposed to foragers had the highest average nectar volumes midway through their lives (Day 3), not when newly open (Figure 1B), even though robbing holes accumulate over time. Third, the sizes of the error bars in Figure 1B clearly indicate that some middle-aged flowers contained exceedingly high volumes of nectar, whereas all newly opened flowers had predictably small volumes. Finally, the presence of robbing holes was not always predictive of a lower nectar volume. As a whole, then, newly opened, unrobbed *C. caseana* flowers should be of predictably low value to foragers (including primary robbers) because they are the only flowers that will reliably contain very little nectar. In contrast, older flowers, even robbed ones, can be bonanzas. 

## 3. Discussion

Plants whose flowers are visited by foragers that exhibit diverse feeding tactics offer an exceptional opportunity for field studies on how mutualism functions in the presence of exploitation. It is possible to quantify the production and depletion dynamics of the rewards that attract and reward both pollinators and nectar larcenists, e.g., [26]. It is also possible to determine why floral visits by foragers that do not transfer pollen are often costly, sometimes neutral, and occasionally beneficial [11,18,20,27]. 

Studies like these, which have become common in recent years, take the plant perspective. Much rarer is work that examines the dynamic from the foragers’ perspective. While extensive attention has been paid to the cognitive ecology of pollinators, including which flowers a pollinator individual should choose and why [28,29,30], the choices faced by floral larcenists are much less clear. It is not known, for example, when or why individual foragers switch between pollinating and larcenous behaviors. Nor is it understood which larcenous behavior they adopt when more than one is available to them. Some answers have begun to emerge, e.g., [11,14,15,16,17,22,31]. However, one question has gone largely unaddressed. Given that some nectar-robbers do adhere to this foraging strategy for prolonged periods, which flowers will they choose, and why? 

A reasonable starting point is to assume that nectar-robbers have at least some information about the relative value of flowers (i.e., most nectar-robbers are experienced foragers), which they then use to choose the most rewarding ones. In this light, it is particularly intriguing to observe flowers within a population that bear multiple robbing holes. A robbing hole is information that a flower has already been fed from. This would seem to be a signal that that flower is not worth a visit. Indeed, plant species in which nectar-robbing reduces seed production are commonly ones in which pollinators avoid such flowers [11]. Should not the robbers skip over robbed flowers as well? Yet, multiple robbing of individual flowers has been recorded in a variety of plant taxa (*Linaria vulgaris*, [32]; *Quassia amara*, [33]; *Arctostaphylos pungens*, [34]; and *Corydalis cavens*, [31]). To our knowledge, this is the first study directed toward understanding the causes of this pattern.

Multiple robbing by bumble bees (with *Bombus mixtus* being the dominant and likely sole robber species at our study sites) is exceptionally common in the wildflower *Corydalis caseana* in the West Elk Mountains of Colorado, USA. Over the three years of this study, 22–70% of all flowers showed evidence of primary nectar robbing, and 22–36% of robbed flowers exhibited more than one hole. High rates of robbing in this species have been noted in other studies as well, conducted in different years and in different populations [35,36]. 

Two fairly straightforward explanations are clearly inadequate to account for multiple robbing in *C. caseana*. First, a new hole might be made only once a primary robber fails to locate an unrobbed flower (we set aside here the possibility that foragers should switch at this point to a strategy other than primary nectar robbing, recognizing that this might occasionally occur [14,16]). We do not have enough information on cognition of *B. mixtus* to know how much information they acquire before deciding to stop searching for an unrobbed flower to visit, if in fact they behave this way. However, the fact that multiply robbed flowers are frequent in this population when well under half the flowers, even on a single stalk, have been robbed a single time does suggest that bees frequently encounter unrobbed flowers. Yet, they rob a previously robbed flower again, rather than choosing to be the first robber of a different flower.

Second, robbing more than once in quick succession might be a tactic a forager adopts in order to fully drain a flower. This seems to be a likely explanation for multiple robbing holes in flowers of another species, *Arctostaphylos pungens* (Ericaceae); its nectar is presented in several deep wells and its primary robbers, solitary *Andrena* bees, move around the flower, quickly drilling several holes that allow access to the nectar (J.L. Bronstein, unpubl. data). The floral structure of *C. caseana* is quite different, with nectar presented in a single nectar spur. Nevertheless, it is possible that a bee might choose to make several access holes in the course of a single visit, especially given that a single robbing event often does not fully drain a *C. caseana* flower [35]. However, we found that in over a third of the multiply robbed flowers, the additional hole(s) had been made on a different day from the first hole. While this does not rule out the possibility that some foragers do drill more than one hole in a single visit, it does exclude the possibility that this is a full explanation for multiple robbing. 

The last possibility we examined is that nectar-robbers are making economically sensible foraging decisions: specifically, that at least some robbed flowers contain more nectar than unrobbed ones do. This seems counterintuitive, given that, as pointed out above, a robbing hole signals an earlier visit. But there are reasons to consider this reasonable in a plant whose flowers last several days and that refill with nectar each day. It is possible, in this case, for a flower with a hole to have been visited by a robber several days earlier, with a subsequent opportunity to replenish. 

In combination with stochastic visitation rates by all floral foragers, the picture that emerges is of a highly variable nectar landscape in which the presence of a robbing hole is not particularly informative. In such a landscape, it might not be worth the time it would take a bee to be highly selective and skip over previously robbed flowers. In our system, however, there is evidence that at least some information is available to the forager: the youngest flowers may not be worth visiting because they have predictably very little nectar. These are also the flowers that are least likely to be robbed, simply because there has been the least time available for robbing to have taken place. Hence, if the bees are unable to directly assess flowers by age—something that we do not know—they might still use the absence of a hole to indicate that a flower that is young and nectar-poor. Indeed, our results indicate that the flowers with the highest nectar volumes are the middle-aged ones. Thus, these results point to the intriguing conclusion that it is worth robbing an older flower again, rather than being the first robber of a young one.

Our results are reinforced by Maloof’s [25,35] studies of *C. caseana* in 1996, near our own site. In that year, a different nectar-robber, the now less-common *B. occidentalis* [37], was its major robber [25,35]. As in our study, nectar was found to accumulate through the life of a flower; the robbing rate approached 60%; robbed flowers contained much less nectar than unrobbed ones on average; and multiple holes were noted on individual flowers. In addition, Maloof [35] found that robbing was more common on 2 to 5-day-old flowers than on first-day flowers, consistent with our argument that the youngest flowers are of lowest value to robbers and might be avoided.

We have relied here on inferences from the damage that nectar-robbers leave behind, rather than observing foraging bouts for the many hours it would take to elucidate their choices directly. Furthermore, very little is known about the cognitive ecology underlying the behavior of these bees, limiting the conclusions we can draw about what they do and do not perceive. It is well documented that bumble bees assess flower quality using multiple sensory modalities and cues and adjust their foraging behavior to maximize foraging efficiency [28,38]. Making additional robbing holes to access nectar would be consistent with what is known about bee cognition. However, it is not clear how sensitive bees are to very small differences in nectar volumes, such as those we have documented between robbed and unrobbed flowers on many days.

A second caveat is that we have assumed that nectar refills in flowers no differently if the flower were drained as if it was visited legitimately (using a capillary tube placed through the floral opening) rather than through a robbing hole. We have no reason to believe that there would be a difference. Other studies showed that nectar replenishment does not differ between flowers that are robbed vs. legitimately visited, e.g., [39]. However, we should note that removing nectar using a capillary tube does not simulate a visit by a pollinator, given that, presumably, no pollen was removed from the flower or deposited on the stigma, and pollination and ovule fertilization can result in a reduction or termination of nectar production (reviewed in [40]). With regard to *C. caseana*, it seems unlikely that robbed flowers would be retained if they did *not* contain nectar. Further, Heiling et al. [18] showed that pollinators visit *C. caseana* flowers regardless of whether or not they have been robbed, using an experiment in which nectar was drained experimentally through an artificial hole. This observation strongly suggests that robbed flowers do refill with nectar. Further experiments will be necessary to address these issues, perhaps ones controlling for age and nectar volume, either in field settings or in the laboratory with artificial flowers.

Third, this study focused on a single population, although across three years. Only one bumble bee species, *Bombus mixtus*, was observed visiting *C. caseana* as a primary nectar-robber during this study cf. [41], although the bumble bee community is relatively rich and other species were observed at different times and sites acting as secondary robbers and as legitimate visitors. We note again, however, that elements of our results are consistent with data from Maloof [25,35] obtained 20 years previously, with a bumble bee species now relatively uncommon at our site. 

Finally, we recognize that a broader perspective on the issue we have addressed here will require integrated study from the forager and plant perspective. Plants are under selection to provision flowers with nectar for pollinators, and the evolutionary dynamics of their interactions require as much attention as we have argued that the forager perspective deserves. Most exciting would be an eco-evolutionary approach that would delve more deeply into the dynamics of the plant—pollinator—robber triad.

What other nectar-robbed plant species might be expected to show multiple robbing holes on a given flower? We can make two predictions. First, flowers in which nectar is held in deep wells are more likely to be multiply robbed. An extreme example is *Aquilegia* spp. (Ranunculaceae), whose flowers exhibit five elongated spurs; robbers and pollinators both must move between these spurs if they are to access all of an individual flower’s nectar. Second, plants with flowers that last several days and that replenish with nectar regularly should be more likely to exhibit signs of multiple robbings than will flowers that are short-lived and/or do not refill. In those systems, a robbing hole is considerably more likely to convey useful information to a visitor. An additional prediction is that the likelihood of multiple robbing should vary according to the taxon of the visitor. Honey bees and hummingbirds are well-documented to avoid revisiting robbed flowers [11]. Bumble bees, in contrast, seem to be less choosy, e.g., [18,42]. Exploring the reason(s) for these differences in selectivity, including cognition, energetics, and life history, would yield important insights.

In conclusion, we can consider the implications of multiple robbing for the general question of how mutualism persists in the face of cheating. For *C. caseana* in particular, it is unlikely to matter if robbing holes are clustered or distributed, since its pollinators visit flowers irrespective of whether they have been robbed [18]. It therefore seems highly unlikely that they would discriminate among robbed flowers based on the number of holes. More generally, though, the clustering of damage might be viewed as a foraging strategy that is adaptive for the forager, but that has the added effect of reducing the cost of cheating to its mutualistic partner. In plant species in which robbing is in fact detrimental, this behavior means that a few flowers will bear the full cost. Other flowers will escape damage entirely. Thus, multiple robbing is a behavior that might serve to align the interests of the partners, a phenomenon increasingly recognized to stabilize mutualism in the face of cheating [2,9,43].

## 4. Materials and Methods

### 4.1. Study System

*Corydalis caseana* (Fumariaceae) is an herbaceous, moderately long-lived perennial occurring in wet meadows, seeps and drainages of the subalpine zone of North America’s mountain west. It grows in dense stands, with stalks reaching 1–1.5 m in height; mature plants bear approximately 20 stalks [25]. Plants begin rapid growth soon after snowmelt and die back in late summer after seeds are shed. This study was conducted from 2014 to 2016 in Washington Gulch (38°95′99.7″ N, 107°03′37.9″ W; 3149.80 m a.s.l.), located in the Elk Mountains of Gunnison County, Colorado, USA. At the study site, it flowers yearly in June and July. Stalks bear numerous terminal racemose, occasionally branching, inflorescences with approximately 5–70 flowers each [25]. The bilaterally symmetrical flowers bear a single nectar spur. The flowers are hermaphroditic. While pollen is readily transferred to the stigmas autogamously, the flowers are only partially self-compatible, with seed set approximately 1.5 times higher in outcrossed flowers; open pollinated flowers produce 5.0 ± 0.8 seeds per fruit [25]. In the West Elk Mountains, bumble bees (*Bombus* spp.) are the most common floral visitors to *C. caseana*. The long-tongued bumble bee *Bombus appositus* is the most common pollinator, responsible for >50% of all visits to the species in some years [25]. Less frequent, more spatially and temporally variable visitors include *Bombus flavifrons*, *B. bifarius*, *B. balteatus*, *B. mixtus*, *B. frigidus*, *B. rufocinctus*, *B. nevadensis* and *B. fervidus*, as well as some non-*Bombus* bees, hummingbirds and butterflies. The guild of more variable bumble bee visitors vary in tongue length from short- to long-tongued.

Floral larceny at the community scale has been well-studied in this region [15,16,17,18,20,25,35,41,44,45,46]. *Corydalis caseana* is consistently and heavily primary robbed in and near this study site; it also experiences high levels of secondary robbing [36]. The nectar spurs of the flowers are long (12–16 mm), making it difficult for some short-tongued bumble bees to access the nectar. Primary nectar-robbers include at least two short-tongued bumble bee species, *Bombus occidentalis* and *B. mixtus*. While *B. occidentalis* is historically the most common nectar-robber on *C. caseana*, and was once responsible for approximately 30% of all floral visits [25], it has become less common in recent years [37]. It was not observed at our study site in 2016 [41], when most of the work reported here was conducted. *Bombus occidentalis* and *B. mixtus* can act not only as primary robbers but also secondary robbers of *C. caseana* [16]. However, switching between these or other foraging tactics is rare [14]. For this reason, we do not address the intriguing question of why primary robbers do not switch to secondary robbing, i.e., to reusing pre-existing holes, or to legitimate visitation. Rather, consistent with our other studies, we assume that individuals only rarely shift tactics within foraging bouts. Exploring the economics underlying these tactics lies beyond the scope of this study (but see [14,16,17]).

Nectar robbing does not damage *C. caseana*’s reproductive or nectar-producing structures and does not interfere with fertilization or seed development. Two studies have found that the effects of robbing on fruit and seed set are neutral [18,35], in large part because its bumble bee pollinators do not avoid robbed flowers [18]. 

### 4.2. Robbing Rates

We recorded population-wide rates of primary nectar robbing, and, among robbed flowers, the number of robbing holes per flower, for three years. Censuses were conducted approximately twice weekly during the month of peak flowering (early July to early August). All flowers on haphazardly chosen stalks were examined. *Corydalis caseana* plants may bear more than one stalk; given the difficulty of identifying separate plants within dense patches, we intentionally avoided censusing stalks that were close neighbors. We sampled 15–20 stalks at each 2014 census date, 18 stalks at each 2015 census date, and 10 stalks at each 2016 census date for a total of 7574 flowers. 

### 4.3. Floral Longevity and Accumulation of Robbing Holes over Time

In mid-July 2016 we tagged nine flowering stalks. As new flowers opened on these stalks, they were given small, inconspicuous, date-specific marks. We revisited these flowers once each day in the late afternoon and recorded the presence and number of robbing holes on each. Flowers were followed until they abscised from the plant.

### 4.4. Nectar Volumes in Open Flowers

To obtain an overview of the “nectar landscape” for flower visitors, we measured nectar volumes from robbed and unrobbed *C. caseana* flowers of unknown age. Using a 2 μL capillary tube, nectar volumes of 70 unrobbed and 26 robbed flowers were measured on 10 July 2015, and nectar volumes in 28 unrobbed and 89 robbed flowers were measured on 26 July 2016. For each sampling date, we compared nectar volumes with a one-sided *t*-test to see if robbed flowers contained less nectar than unrobbed ones.

### 4.5. Nectar Production

We measured *C. caseana* nectar production rate as a function of flower age in 2016. Bags were placed around nine haphazardly chosen stalks bearing buds; all open flowers were carefully removed. Each morning, each bag was opened. Three newly opened flowers within each bag were removed from the plant; nectar was drained and the volume measured using a 2 μL capillary tube. All other open flowers were given a date-specific mark. The bags were then replaced. The following morning, we removed the bags and recorded both the number of flowers marked the previous day (now Day 2 flowers) that remained open, as well as any newly opened flowers, to which we gave a date-specific mark. We then removed three Day 2 flowers and three Day 1 flowers and recorded their nectar volume as described above. We proceeded this way, day by day, until no flowers remained within the bags. In this way, we determined nectar production rate over the life of a flower (n = 200 flowers).

These data on nectar production rate provide no information on whether *C. caseana* flowers refill with nectar once drained. To determine this, we used another manipulative experiment. Twenty stalks were bagged, as described above, and all open flowers were removed. Then, each day, we removed one newly open (Day 1) flower and measured its nectar volume; a second Day 1 flower was marked but its nectar was only measured on Day 2; and a third flower’s nectar was drained and measured on Day 1, and drained and measured again on Day 2. If nectar is replenished after draining, then the third flower was expected to contain nectar on Day 2. If replenishment brings nectar volumes up to the level they would contain if they had not been visited, then the flowers drained on both Days 1 and 2 would have produced, in total, the same amount of nectar as the flowers sampled only on Day 2. A two-sided *t*-test was used to test this prediction.

### 4.6. Tests of Hypotheses

Robbing rate data from 2014–2016 were used to test Hypothesis 1 (multiple holes start accumulating in flowers only once primary robbers have attacked every flower in the population once). We compared the proportion of robbed flowers in the population to the proportion of those robbed flowers exhibiting more than one robbing hole. If multiple holes are frequent when large numbers of flowers lack a single robbing hole, this hypothesis can be rejected. Without more information on bumble bee cognition and searching behavior, we cannot predict the point at which bees might abandon a search for an unrobbed flower and shift to using a previously robbed one. However, if flowers with multiple holes are frequent even when unrobbed flowers are abundant in the population, this would strongly suggest that bees have no difficulty locating unrobbed ones, but are choosing to re-rob robbed flowers instead.

Floral longevity and hole-accumulation data from 2016 were used to test Hypothesis 2 (individual robbers make multiple holes during a single visit to a flower). We did not observe foraging behavior of enough individual bees to test this hypothesis directly. Rather, we reasoned that a conservative test of this hypothesis would be as follows. If all multiple robs were attributable to individual foraging visits, then all should accumulate during a single day in the life of the flower. If a large proportion of multiple robs accumulate on different days, then the hypothesis that multiple robs are always the outcome of behaviors exhibited during a single flower visit can be rejected. This does not rule out the possibility that *some* multiple robs are the outcome of individual visits. Nor can it distinguish between multiple robs made in a single visit from those taking place on the same day by the same or different bees during different visits. However, if multiple holes typically accumulate on different days across the life of the flower, it does rule out the possibility that *all* of them are the outcome of single foraging visits. 

Finally, Hypothesis 3 proposes that the dynamics of nectar replenishment and accumulation of robbing holes make older flowers more profitable for primary robbers to visit than younger ones that have never been robbed. To test this, we combined data on floral longevity, accumulation of robbing holes in exposed flowers, nectar volumes of open flowers of known age, and nectar production rates in bagged flowers. Hypothesis 3 can be rejected if nectar volumes are highest in the youngest flowers that have not been primary robbed.

All statistics were carried out using JMP Pro 16.2.0.

## Figures and Tables

**Table 1:** Date of appearance of multiple holes in individual flowers in July 2016. Overall, 36.4% of second, third, and fourth holes were made in flowers one or more days after the first hole had been made. Thus, at least these multiple holes could not have been made during a single primary robbing visit to a flower.

	2 Holes	3 Holes	4 Holes
Made same day	14	5	2
Made different day	8	3	1

## Data Availability

Data are available via Dryad at DOI: 10.5061/Dryad.W3r22811f.

## References

[1] Bronstein J.L., Bronstein J.L. (2015). The study of mutualism. Mutualism.

[2] Jones E.I., Afkhami M.E., Akcay E., Bronstein J.L., Bshary R., Frederickson M.E., Heath K.D., Hoeksema J.D., Ness J.H., Pankey M.S. (2015). Cheaters must prosper: Reconciling theoretical and empirical perspectives on cheating in mutualism. Ecol. Lett..

[3] Weyl E.G., Frederickson M.E., Yu D.W., Pierce N.E. (2010). Economic contract theory tests models of mutualism. Proc. Natl. Acad. Sci. USA.

[4] Kiers E.T., Denison R.F., Kawakita A., Herre E.A. (2011). The biological reality of host sanctions and partner fidelity. Proc. Natl. Acad. Sci. USA.

[5] Frederickson M.E. (2017). Mutualisms are not on the verge of breakdown. Trends Ecol. Evol..

[6] West S.A., Cooper G.A., Ghoul M.B., Griffin A.S. (2021). Ten recent insights for our understanding of cooperation. Nat. Ecol. Evol..

[7] Bronstein J.L. (2001). The exploitation of mutualisms. Ecol. Lett..

[8] Porter S.S., Faber-Hammond J., Montoya A.P., Friesen M.L., Sackos C. (2019). Dynamic genomic architecture of mutualistic cooperation in a wild population of *Mesorhizobium*. ISME J..

[9] Batstone R.T., Burghardt L.T., Heath K.D. (2022). Phenotypic and genomic signatures of interspecies cooperation and conflict in naturally occurring isolates of a model plant symbiont. Proc. R. Soc. Lond. B.

[10] Vidal M.C., Agarwal R., Segraves K.S. (2024). Coevolution and dependency influence resistance of mutualists to exploitation. Front. Ecol. Evol..

[11] Irwin R.E., Bronstein J.L., Manson J.S., Richardson L. (2010). Nectar robbing: Ecological and evolutionary perspectives. Annu. Rev. Ecol. Evol. Syst..

[12] Inouye D.W. (1980). The terminology of floral larceny. Ecology.

[13] Richardson L.L., Bronstein J.L. (2012). Reproductive biology of pointleaf manzanita (*Arctostaphylos pungens*) and the pollinator-nectar robber spectrum. J. Pollinat. Ecol..

[14] Bronstein J.L., Barker J.L., Lichtenberg E.M., Richardson L.L., Irwin R.E. (2017). The behavioral ecology of nectar robbing: Why be tactic constant?. Curr. Opin. Insect Sci..

[15] Lichtenberg E.M., Irwin R.E., Bronstein J.L. (2018). Costs and benefits of alternative food handling tactics help explain facultative exploitation of pollination mutualisms. Ecology.

[16] Lichtenberg E.M., Irwin R.E., Bronstein J.L. (2020). Bumble bees are constant to nectar robbing behavior despite low switching costs. Anim. Behav..

[17] Lichtenberg E.M., Richman S.K., Irwin R.E., Bronstein J.L. (2020). Competition for nectar resources does not affect bee foraging tactic constancy. Ecol. Entomol..

[18] Heiling J.M., Ledbetter T.A., Richman S.K., Ellison H.K., Bronstein J.L., Irwin R.E. (2018). Why are some plant–nectar robber interactions commensalisms?. Oikos.

[19] Rojas-Nossa S.V., Sánchez J.M., Navarro L. (2021). Nectar robbing and plant reproduction: An interplay of positive and negative effects. Oikos.

[20] Maloof J.E., Inouye D.W. (2000). Are nectar robbers cheaters or mutualists?. Ecology.

[21] Hou Q.-Z., Ehmet N., Chen D.-W., Wang T.-H., Xu Y.-F., Ma J., Sun K. (2021). Corolla abscission triggered by nectar robbers positively affects reproduction by enhancing self-pollination in *Symphytum officinale* (Boraginaceae). Biology.

[22] Barker J.L., Dornhaus A., Bronstein J.L., Muth F. (2018). Learning about larceny: Experience can bias bumble bees to rob nectar. Behav. Ecol. Sociobiol..

[23] Richman S.K., Barker J.L., Baek M., Papaj D.R., Irwin R.E., Bronstein J.L. (2021). The sensory and cognitive ecology of nectar robbing. Front. Ecol. Evol..

[24] Baek M., Bish S.E., Giebink N.W., Papaj D.R. (2023). The interplay of experience and pre-existing bias in nectar-robbing behavior by the common eastern bumble bee. Behav. Ecol. Sociobiol..

[25] Maloof J.E. (2000). Reproductive biology of a North American subalpine plant: *Corydalis caseana* A. Gray ssp. *brandegei* (S. Watson) G. B. Ownbey. Plant Species Biol..

[26] Luo E.Y., Ogilvie J.E., Thomson J.D. (2014). Stimulation of flower nectar replacement by removal: A survey of eleven animal-pollinated plant species. J. Pollinat. Ecol..

[27] Irwin R.E., Brody A.K., Waser N.M. (2001). The impact of floral larceny on individuals, populations, and communities. Oecologia.

[28] Chittka L., Thomson J.D. (2001). Cognitive Ecology of Pollination: Animal Behavior and Floral Evolution.

[29] Chittka L. (2022). The Mind of a Bee.

[30] Buchmann S.L. (2023). What a Bee Knows.

[31] Olesen J.M. (1996). From näiveté to experience: Bumblebee queens (*Bombus terrestris*) foraging on *Corydalis cava* (Fumariaceae). J. Kans. Entomol. Soc..

[32] Stout J.C., Allen J.A., Goulson D. (2000). Nectar robbing, forager efficiency and seed set: Bumblebees foraging on the self incompatible plant *Linaria vulgaris* (Scrophulariaceae). Acta Oecol..

[33] Roubik D.W., Holbrook N.M., Parra G. (1985). Roles of nectar robbers in the reproduction of a tropical treelet, *Quassia amara* (Simaroubaceae). Oecologia.

[34] Eliyahu D., McCall A.C., Lauck M., Trakhtenbrot A. (2015). Florivory and nectar-robbing perforations in flowers of pointleaf manzanita *Arctostaphylos pungens* (Ericaceae) and their effects on plant reproductive success. Arthropod-Plant Interact..

[35] Maloof J.E. (2001). The effects of a bumble bee nectar robber on plant reproductive success and pollinator behavior. Am. J. Bot..

[36] Irwin R.E., Maloof J.E. (2002). Variation in nectar robbing over time, space, and species. Oecologia.

[37] Janousek W.M., Douglas M.R., Cannings S., Clément M.A., Delphia C.M., Everett J.G., Hatfield R.G., Keinath D.A., Koch J.B.U., McCabe L.M. (2023). Recent and future declines of a historically widespread pollinator linked to climate, land cover, and pesticides. Proc. Natl. Acad. Sci. USA.

[38] Goulson D. (1999). Foraging strategies of insects for gathering nectar and pollen, and implications for plant ecology and evolution. Perspect. Plant Ecol. Evol. Syst..

[39] Ye Z.M., Jin X.F., Wang Q.F., Yang C.F., Inouye D.W. (2017). Nectar replenishment maintains the neutral effects of nectar robbing on female reproductive success of *Salvia przewalskii* (Lamiaceae), a plant pollinated and robbed by bumble bees. Ann. Bot..

[40] Ordano M., Ornelas J.F. (2004). Generous-like flowers: Nectar production in two epiphytic bromeliads and a meta-analysis of removal effects. Oecologia.

[41] Ledbetter T.A., Richman S.K., Irwin R.E., Bronstein J.L. (2022). What are the reproductive consequences of losing a nectar-robber?. J. Pollinat. Ecol..

[42] Richardson S.C. (2004). Are nectar-robbers mutualists or antagonists?. Oecologia.

[43] Brockhurst M.A., Cameron D.D., Beckerman A.P. (2024). Fitness trade-offs and the origins of endosymbiosis. PLoS Biol..

[44] Richman S.K., Irwin R.E., Bronstein J.L. (2017). Foraging strategy predicts foraging economy in a facultative secondary nectar robber. Oikos.

[45] Richman S.K., Irwin R.E., Nelson C.J., Bronstein J.L. (2017). Facilitated exploitation of pollination mutualisms: Fitness consequences for plants. J. Ecol..

[46] Richman S.K., Irwin R.E., Bosak J.T., Bronstein J.L. (2018). Consequences of secondary nectar robbing for male components of plant reproduction. Am. J. Bot..

